# Clinical implications of a very late presentation in myasthenia gravis: real world experience in a tertiary center

**DOI:** 10.1055/s-0046-1824432

**Published:** 2026-06-23

**Authors:** André Aires Fernandes, Luís Braz, Goreti Nadais, Fernando Silveira, Maria João Pinto

**Affiliations:** 1 Unidade Local de Saúde de São João, Serviço de Neurologia, Porto, Portugal.; 2 Universidade do Porto, Faculdade de Medicina, Departamento de Neurociências Clínicas e Saúde Mental, Porto, Portugal.

**Keywords:** Autoimmune Diseases, Myasthenia Gravis, Acetylcholine, Disease Onset, Prognosis

## Abstract

**Background:**

Myasthenia gravis (MG) is an immune-mediated disease with a bimodal incidence: early-onset (< 50 years, predominantly female) and late-onset (> 50 years, predominantly male). Recently, a subgroup with very late-onset (≥ 65 years) has been described; however, its clinical relevance concerning disease course and prognosis remains uncertain.

**Objective:**

To evaluate the potential clinical implications of very late presentations of MG.

**Methods:**

The present is a retrospective cohort study comprising all patients diagnosed with MG currently followed up in the Neuromuscular Disorders Unit of a tertiary center. Clinical and paraclinical parameters including number of hospitalization and current regime were evaluated.

**Results:**

A total of 92 patients were included, 51 (55.4%) of whom were female. Concerning the age of onset, 23 (25.6%) patients were classified as very late-onset. In order to better understand the clinical implications of a later onset, we compare the very late-onset subgroup to the late-onset subgroup. Gender distribution and number of patients with minimal manifestation status or better did not statistically differ between these subgroups. Myasthenic crisis and refractoriness were more frequent in the late-onset subgroup compared with very late-onset, but no statistical difference was found ( *p* = 0.221 and *p* = 0.650 respectively). We did not find statistically significant differences in acetylcholine receptor antibody titers between the groups. Concomitant autoimmune diseases were found in 14 (15.2%) patients, comprising three patients with late-onset and three patients with very late-onset MG.

**Conclusion:**

Our study suggests that the recently established subgroup of very late-onset may not be clinically distinct from late-onset MG, as these patients seem to exhibit a similar clinical trajectory.

## INTRODUCTION

Myasthenia gravis (MG) is an immune postsynaptic neuromuscular junction disorder characterized by fatigable muscle weakness. 1 2 3 It is heterogeneous and often classified by age at onset, clinical profile, autoantibody status, and thymus pathology. 1 4 Traditionally, MG's bimodal onset is divided at 50 years, but an increased incidence in those ≥ 65 years has led to the proposal of a very late-onset subgroup. 4 5 The importance of the age-of-onset classification lies in the clinical heterogeneity associated with older presentations. 4 According to literature, later-onset MG occurs slightly more frequently in males, patients are more likely to be seropositive for autoantibodies against the acetylcholine receptor (AChR) antibody and less likely to have a thymoma. 1 4 6 7 Reports conflict on whether very late-onset MG is clinically distinct, with some studies showing a poorer prognosis and others indicating similar or milder severity, lower requirement for immunosuppressive therapies and lower rates of refractoriness to first-line drugs compared to late-onset cases. 4 5 Randomized controlled trials, although essential for establishing the efficacy and safety of new pharmacological interventions, are limited by their controlled conditions, restricted inclusion criteria, and relatively short duration, and may, therefore, fail to capture long-term adverse effects associated with sustained use of these therapies in a real-world population of MG patients.

The present study aims to characterize the clinical course of patients with MG according to age at disease onset, with particular emphasis on comparing very-late onset and late-onset patients in terms of disease control, occurrence of myasthenic crises, hospitalizations, and response to immunosuppressive therapy.

## METHODS

### Study design

We conducted a retrospective cohort study of all patients diagnosed with MG and followed up in the Neuromuscular Disorders unit of Unidade Local de Saúde de São João between January 1st, 2000, and May 31st, 2023. Diagnosis was based on fatigable skeletal muscle weakness plus at least one of the following:

- positive autoantibodies against AChR or muscle-specific tyrosine kinase (MuSK);
- repetitive nerve stimulation at 3 Hz showing a gradual decline in compound muscle action potential;
- increased jitter in single-fiber stimulation; or
- clear improvement with symptomatic or immunosuppressive treatment.

Patients were considered double-seronegative if they tested negative for both anti-AChR and anti-MuSK antibodies. No patients in our cohort were diagnosed solely based on the fourth, less specific diagnostic criterion; all diagnoses met at least one of the first three, more specific criteria. All the double-seronegative patients fulfilled neurophysiological criteria for MG, and the diagnosis was further supported by the clinical course, including the absence of disease generalization and a positive response to immunosuppressive therapy.

### Data collection

We collected demographic (sex, current age) and clinical data, including age of onset, disease duration, and comorbid autoimmune diseases. Patients were divided according to the Task Force of the Myasthenia Gravis Foundation of America (MGFA) classification: ocular MG when symptoms were restricted to the ocular muscles for at least 3 months; or generalized MG defined as involving weakness of the limb, trunk, dysphagia, dysphonia, or respiratory muscles. 8 Based on onset age, patients were classified as juvenile-onset (< 18 years), early-onset (18–49 years), late-onset (50–64 years), and very late-onset (≥ 65 years). 4 Serological tests on autoantibodies against AChR and MuSK were performed. Thymic status was evaluated either through chest computed tomography (CT) or postoperative pathology.

Refractory disease was defined as failure to improve after corticosteroids and at least 2 other immunosuppressive agents, used in adequate doses for an adequate duration (minimum of 6 months), with persistent symptoms or side effects that led to drug withdrawal, as defined by the patient and the physician. 8

We included information on chronic immunosuppression and myasthenic crises management. Response to treatment was evaluated using the MGFA Task Force Post-Intervention Status classification, as follows: Minimal Manifestation Status (MMS) - the patient has no symptoms or functional limitations from MG but has some weakness on examination of some muscles; Remission - weakness of eyelid closure is accepted, but there is no weakness of any other muscle on careful examination and the patient is asymptomatic. 8 All MG exacerbations leading to hospitalization were evaluated, including episodes of impending and definite myasthenic crises according to the international consensus guidance for management of myasthenia gravis. 8

Additionally, we conducted a comparative analysis of clinical, immunological, and therapeutic attributes within a subset of individuals exhibiting very-late-onset MG, compared with those of the late-onset MG subgroup.

### Statistical analysis

Statistical analysis was performed using the IBM SPSS Statistics for Windows (IBM Corp.) software, version 27.0. We presented categorical variables in absolute and relative frequency and continuous variables with a normal distribution as mean ± standard deviation (SD). The patients were classified based on the age of onset. Statistical analyses were performed using appropriate parametric or non-parametric tests according to data distribution. Normality of continuous variables was assessed using the Shapiro-Wilk test. Continuous variables with a normal distribution are presented as mean ± SD and were compared using one-way analysis of variance (ANOVA), with homogeneity of variances assessed using Levene's test. Continuous variables with a non-normal distribution are presented as median and interquartile range and were compared using the Mann-Whitney U test. Categorical variables are presented as absolute numbers and percentages and were compared using the Chi-squared or Fisher's exact tests, as appropriate. All statistical tests were two-tailed, and statistical significance was defined as a *p* -value < 0.05.

### Ethics and consent to participate

The current study was conducted according to the principles of the Declaration of Helsinki, and patient written consent was obtained. The study was approved by the Ethics Committee of Unidade Local de Saúde de São João (no. 320/23).

## RESULTS

A total of 92 patients were included, 51 (55.4%) of whom were female, with a mean age of 59.9 ± 18.0 years. We summarized the demographic and clinical data in Table 1 . Regarding age of onset, 7 (7.6%) patients were considered juvenile, 42 (45.8%) early-onset, and 20 (21.7%) late-onset. The very late-onset subgroup had 23 (25%) patients, 4 of whom were diagnosed > 80 years. There were 66 (71.7%) patients positive for anti-AChR antibodies, 3 (3.3%) for anti-MuSK antibodies, and the remaining 23 (25.0%) were double-seronegative. Among the double-seronegative patients, the majority (59.1%; 13/22) had ocular MG. A generalized form of MG was observed in 76.1% (n = 70) of patients, while 23.9% (n = 22) had only ocular impairment. Thymectomy was performed in 37 (40.2%) patients, 15 of whom presented with thymoma. Concomitant autoimmune diseases (AIDs) were found in 14 (15.2%) patients ( Table 2 ). Autoimmune comorbidity was positively associated with refractoriness ( *p* = 0.026). Additionally, although not statistically significant, a lower proportion of patients achieving minimal manifestations status or better was observed in the subgroup with AIDs ( *p* = 0.075). Patients with concurrent AIDs showed a tendency for adverse effects due to immunosuppressive/immunomodulatory therapies ( *p* = 0.066).

**Table 1:** Clinical and paraclinical characteristics of the MG patients

Gender (female)			51 (55.4%)
Mean age (last evaluation)			60.8 ± 17.9
Onset	Juvenile	7 (7.6%)
	Early-onset		42 (45.6%)
	Late-onset		20 (21.7%)
	Very late-onset		23 (25.6%)
Hospitalization diagnosis (yes)			53 (57.6%)
Time of disease (mean)			13.7 ± 10.4
Phenotype	Ocular	22 (23.9%)
	Generalized		70 (76.1%)
Antibody status	Anti-AchR	66 (71.7%)
	Anti-MuSK		3 (3.3%)
	Double seronegative		22 (23.9%)
Thymoma			15 (16.3%)
Thymectomy			37 (40.2%)
Refractory MG			19 (20.7%)
Hospitalization due to exacerbation		22 (23.9%)
	1	10
	2–5	10
	> 5	3
Concomitant autoimmune disease*		14 (15.2%)
	Thyroid autoimmunity	6
	Autoimmune gastritis	2
	Celiac disease	1
	Vitiligo	1
	Psoriasis	1
	Rheumatoid arthritis	2
	Systemic lupus erythematosus	2
	Glomerulonephritis	1
	Chronic inflammatory demyelinating polyneuropathy	1

Abbreviations: Anti-AchR, antibodies against the acetylcholine receptor; Anti-MuSK, antibodies against muscle-specific tyrosine kinase; MG, myasthenia gravis.

Note: *Three patients presenting more than one autoimmune disorder.

**Table 2:** Clinical characterization of patients with concomitant autoimmune disease

	Concomitant autoimmune disease		*p*
	Yes	No	
Female	11 (78.6%)	40 (51,3%)	0.059
Late-onset	6 (42.9%)	37 (47.4%)	1
Generalized myasthenia gravis	12 (85.7%)	58 (74.4%)	0.359
Seropositive myasthenia gravis	11 (78.6%)	58 (74.4%	0.794
Myasthenia gravis exacerbations	5 (35.7%)	17 (21.8%)	0.261
Thymoma	3 (21.4%)	12 (15.4%)	0.573
Refractory myasthenia gravis	6 (42.9%)	10 (16.7%)	0.026
Drugs side effects (yes)	3 (21.4%)	5 (6.4%)	0.066
Minimal manifestation status or better	9 (64.3%)	65 (84.4%)	0.075

At the last evaluation, 8 (8.4%) patients fulfilled criteria for complete remission, and 66 (71.7%) patients were classified as having minimal manifestations, 19 (20.7%) of whom were solely under symptomatic treatment with pyridostigmine.

Regarding immunosuppressive and immunomodulatory therapies, low-dose prednisolone (median: 5mg; IQR: 5–13) was used in 37 (40.2%) individuals, including 14 patients in monotherapy. Azathioprine was used as the preferred corticosteroid-sparing agent (n = 26; 28.3%), followed by methotrexate (n = 4; 4.3%) and mycophenolate mofetil (n = 1; 1.1%). Nineteen (20.7%) cases were classified as drug-refractory MG. In this subgroup, rituximab was the most commonly used alternative therapy (11/19), with rituximab plus intravenous human immunoglobulin (IVIg) in 4 patients, and a combination of rituximab, IVIg, and azathioprine in 1 patient. Three patients are currently under periodic IVIg infusion as monotherapy, and two patients are under efgartigimod, a neonatal Fc receptor blocker. One patient with anti-MuSK-positive MG was submitted to autologous bone marrow transplant, achieving total remission of symptoms at 10 months of follow-up ( Table 3 ).

**Table 3:** Actual therapeutic regimens

None	8 (8.7%)
Symptomatic treatment alone	19 (20.7%)
Low-dose oral corticosteroid monotherapy	14 (15.2%)
Azathioprine	29 (31.5%)
Rituximab	9 (9.8%)
Methotrexate	4 (4.3%)
Regular IVIg infusion	4 (4.3%)
Efgartigimod	2 (2.2%)
Mycophenolate mofetil	1 (1.1%)
IVIg + rituximab	1 (1.1%)
IVIg + azathioprine	1 (1.1%)
Bone marrow autologous transplant	1 (1.1%)

Abbreviation: IVIg, intravenous immunoglobulin.

During the follow-up period, eight patients exhibited side effects due to immunosuppressive agents, leading to their discontinuation, including two patients who were intolerant to both azathioprine and methotrexate.

The mean disease duration was 12.7 ± 10.4 years, during which 22/92 patients suffered from at least one myasthenic crisis resulting in hospitalization. Intravenous human immunoglobulin was the preferred treatment used during myasthenic crises, having been administered 39 times, followed by plasmapheresis on 3 crises, and both therapies were used concurrently 4 times.

The overall annualized hospitalization rate was 0.04 (n = 92). Three deaths were registered during follow-up, affecting the very late-onset (n = 2) and the late-onset (n = 1) subgroups. One death was attributable to a fatal complication of the myasthenic crisis, one due to metastatic cancer, and one of undetermined etiology. We found that the seropositive subgroup (AChR or MusK) had a higher hospitalization rate ( *p* = 0.018).

### Comparison of clinical characteristics and prognosis between the late and very late-onset subgroups

We compared the late (50–64 years) and very late-onset (≥ 65 years) subgroups ( Table 4 ). Gender distribution, thymoma incidence, and the number of myasthenic exacerbations leading to hospitalization did not differ significantly between these subgroups ( *p* = 0.395, *p* = 0.650, and *p* = 0.329, respectively).

We did not find statistically significant differences in acetylcholine receptor (AChR) antibody titers between the groups ( *p* = 0.601) or in relation to prognosis (p = 0.571), as assessed by achievement of minimal manifestation status ( Table 5 ).

**Table 4:** Clinical and paraclinical comparison between very-late onset and late-onset patients

		Late-onset (n = 20)	Very late-onset (n = 23)	*p*
Gender (male)		13	12	0.395
Hospitalization at diagnosis (yes)		15	18	0.801
Phenotype	Ocular	8	8	0.724
	Generalized	12	15	
Thymoma		3	2	0.650
Myasthenic exacerbation		6	4	0.329
Minimal manifestation status or better		18	17	0.247
Symptomatic treatment solely		5	5	0.563
Refractory myasthenia gravis		3	2	0.650
Drugs side effects (yes)		1	2	1
Concomitant autoimmune disease		3	3	1

**Table 5:** Assessing AchR antibody and Anti-MuSK seropositivity in late-onset and very late-onset groups

	Late-onset (n = 20)	Very late-onset (n = 23)	*p*
Seropositive MG	13* (65.0%)	18 (78.3%)	0.470
Anti-AchR titers (median)	4.71 (0.55–263.30)	6.39 (1.04–29.93)	0.601

Abbreviations: Anti-AchR, antibodies against the acetylcholine receptor; MG, myasthenia gravis; anti-MuSK, antibodies against the muscle-specific tyrosine kinase.

Note: *Including one patient with anti-MuSK antibodies.

Generalized MG was reported by 27 patients and did not differ between subgroups ( *p* = 0.724). Additionally, we did not find any statistical difference in response to treatment, including the proportion of patients with minimal manifestations or better status, and the frequency of side effects of immunosuppressive agents ( *p* = 0.247 and *p* = 0.554 respectively). At the last evaluation, we found a higher proportion of very late-onset patients solely treated with pyridostigmine compared with late-onset patients ( *p* = 0.563). We identified 5 patients who met criteria for refractory MG: 3 with late-onset and 2 with very late-onset presentations.

## DISCUSSION

The ongoing increase in patients presenting with late- and very-late-onset MG underscores the need for a better understanding of the clinical implications of later presentations. In our cohort, late-onset and very late-onset cases accounted for 22.8% and 23.9% of the included patients, respectively. Notably, data from the largest cohort studies 4 9 indicate a prevalence of late-onset presentation ranging from 24.2 to 35.6%, and very late-onset from 13.9 to 45.2%, highlighting the considerable variability inherent to the populations studied. In fact, these studies 4 9 show that late- and very-late-onset presentations of MG are common and may even occur more frequently than early-onset cases. This trend may reflect greater longevity, improved disease recognition, or underlying immunological factors; however, these remain speculative hypotheses and are not directly supported by data from our study. Moreover, we observed a male predominance in the population with late and very late-onset MG. 5 10 11 12 We did not find any statistically significant differences between the very late-onset and late-onset groups, namely regarding gender distribution, thymoma incidence, the occurrence of impending crisis/myasthenic crisis, and concomitant autoimmune disease. Recently, a large retrospective cohort study conducted by Tang et al. suggested that very late-onset MG had a poorer prognosis than early- and late-onset MG, with a lower percentage of patients achieving minimal manifestations or better. 5 However, our study did not corroborate these findings. Indeed, we found that very late-onset and late-onset MG patients had similar treatment responses and prognoses, including the number of serious exacerbations requiring hospitalization, as previously documented. 11 13

Several factors may explain the discrepancies between our findings and previous reports on very-late-onset MG. In the Chinese cohort, the proportion of thymoma among very-late-onset patients was 10.1%, compared to 8.7% in our study. 5 The distribution of autoantibodies also differed: 62.9% of patients in the Chinese cohort were anti-acetylcholine receptor positive and 3.8% anti-MuSK positive, whereas in our cohort 78.3% were anti-acetylcholine receptor positive and none were anti-MuSK positive. 5 Additionally, very-late-onset patients comprised 13.9% of the Chinese cohort versus 25% in our study. 5 Regarding treatment, differences between our cohort and the Chinese cohort may also have influenced outcomes. Regular IVIg infusions were used in 2 patients (8.7%) in our cohort versus none in the Chinese cohort, while rituximab was administered to 1 patient (4.3%) in our study compared to 2.3% in the Chinese cohort. 5 Low-dose prednisone was used in 60.1% of our patients, versus 36.5% in the Chinese cohort 5 Moreover, genetic differences between European and non-European populations may influence disease presentation and progression and should be considered when comparing outcomes across cohorts. 5 These differences in treatment strategies, together with variations in antibody profiles, thymoma prevalence, and population genetics, highlight the heterogeneity that may contribute to differences in prognosis between studies.

The present study spans a long time period during which treatment strategies for MG evolved considerably, leading to heterogeneity in therapeutic approaches that may have influenced outcomes and should be taken into account when interpreting the results. Although the first reported case of rituximab use in MG was in 1999, the first systematic review was only published in 2014, with additional meta-analyses appearing later; accordingly, rituximab only began to be used more frequently during the second half of the 2010s. 14 15 16 17 More recently, efgartigimod was approved at our hospital in 2024, which explains why only two patients in our cohort were under it. 18 Furthermore, suboptimal disease control prior to treatment initiation may have contributed to patients being undertreated for longer periods, potentially leading to poorer responses.

Given that very-late-onset patients in our cohort received immunosuppressive therapy less frequently than late-onset patients, this may explain why a higher proportion were treated with pyridostigmine for the symptomatic control of mild symptoms.

We observed a higher hospitalization rate among seropositive patients ( *p* = 0.018). This is consistent with previous reports showing that seropositivity in MG, particularly anti-AChR positivity, is associated with a more severe disease course, a higher risk of exacerbations, and increased hospitalizations, whereas double-seronegative patients tend to experience fewer exacerbations and generally have better outcomes. 19 20 21

Although no statistically significant differences were observed between late and very late presentations, the limited sample size and lack of multivariate analysis mean that these results should be interpreted cautiously and do not exclude the possibility of clinically relevant differences. Nevertheless, the absence of clear differences suggests that similar follow-up schedules and therapeutic approaches may be applied to both groups in clinical practice.

The current study has some limitations that should be acknowledged. We must consider the retrospective nature of the study a significant limitation, as it depends on the quality of clinical records. Not all information is available in hospital files, including details on mild adverse reactions. The use of disease severity scores, fatigue, and quality-of-life scales could provide more detailed information on functional outcomes and symptom control. Myasthenia gravis-specific outcome measures, including Myasthenia Gravis Activities of Daily Living (MG-ADL), Quantitative Myasthenia Gravis (QMG), and Myasthenia Gravis Composite (MGC), were not systematically collected at all visits due to time constraints in routine clinical practice and the retrospective nature of the study, which represents a relevant limitation. Other antibodies, beyond anti-AChR and anti-MuSK, were not systematically assessed. The relatively high proportion of double-seronegative patients in our cohort represents an additional limitation, as seronegative MG may encompass heterogeneous disease mechanisms and may limit comparability with cohorts enriched for antibody-positive cases. Additionally, all patients were followed solely in a tertiary center, which may introduce selection bias, and very severe cases may not have reached our center, potentially resulting in survival bias. Diagnostic and therapeutic practices evolved over the 23-year study period, which may have influenced outcomes. Given the relatively small sample size and the absence of control for potential confounders such as sex, autoantibody status, and disease duration, the lack of statistically significant differences between groups may reflect limited statistical power and the possibility of a type-II error rather than a true absence of clinically meaningful differences.

In conclusion, our findings suggest that the recently established subgroup of very late-onset (≥ 65 years) MG may not be clinically relevant, as these patients exhibit a clinical trajectory similar to that of late presentations (50–64 years).
